# Looking on the (B)right Side of Life: Cognitive Ability and Miscalibrated Financial Expectations

**DOI:** 10.1177/01461672231209400

**Published:** 2023-11-10

**Authors:** Chris Dawson

**Affiliations:** 1 University of Bath, UK

**Keywords:** unrealistic optimism, cognitive ability, decision-making

## Abstract

It is a puzzle why humans tend toward unrealistic optimism, as it can lead to excessively risky behavior and a failure to take precautionary action. Using data from a large nationally representative U.K. sample (N=36,312), our claim is that optimism bias is partly a consequence of low cognition—as measured by a broad range of cognitive skills, including memory, verbal fluency, fluid reasoning and numerical reasoning. We operationalize unrealistic optimism as the difference between a person’s financial expectation and the financial realization that follows, measured annually over a decade. All else being equal, those highest on cognitive ability experience a 22% (53.2%) increase in the probability of realism (pessimism) and a 34.8% reduction in optimism compared with those lowest on cognitive ability. This suggests that the negative consequences of an excessively optimistic mindset may, in part, be a side product of the true driver, low cognitive ability.

You can avoid reality, but you cannot avoid the consequences of avoiding reality.

—Ayn Rand

## Introduction

Unrealistic optimism or optimism bias—the tendency for individuals to overestimate the chance of favorable outcomes occurring and underestimate the chance of bad (Weinstein, 1980)—has been found to be one of the most pervasive human traits across many domains (Sharot, 2011). For instance, research has shown that individuals tend to underestimate the likelihood of developing a drinking problem or getting divorced (Weinstein, 1980) and to overestimate their future earnings (Dawson, 2017) and how long they are going to live (Puri & Robinson, 2007). Our established tendency toward unrealistic optimism poses an evolutionary puzzle as normative models of human judgment, like expected utility theory, suggest unbiased assessments of probabilities are advantageous. Like any other judgmental bias, optimism bias distorts the decision-making process, leading to systematic decision errors, increased rash and risky behavior (de Meza et al., 2019) and a failure to take precautionary measures (Dillard et al., 2009).

Explanations of this puzzle have tended to focus on the idea that beliefs are self-serving and are “chosen” to fulfill certain psychological needs (S. E. Taylor & Brown, 1988). More specifically, research has pointed to the immediate affective benefits of unrealistic optimism, such as enhanced psychological well-being and self-esteem (Pyszczynski et al., 1987), improvements in our ability to cope with negative feedback (S. E. Taylor & Brown, 1988), and the ability to savor in our future successes (Brunnermeier & Parker, 2005; Loewenstein, 2006). Indeed, the motivation to maintain an optimistic view of the future can be significant, with the anticipation of unpleasant events having an equally, if not greater, impact on distress levels than the event itself (Berns et al., 2006; Nosarti et al., 2002).

While these affective benefits are thought to provide the incentives for unrealistic optimism, research has also highlighted the channels through which optimism bias can be maintained in the presence of reality and constant feedback from our environment. For instance, the literature has provided an abundance of empirical evidence on the phenomenon of asymmetric information integration—the idea that humans are better at integrating positive information into their beliefs than negative information (Drobner, 2022; Eil & Rao, 2011; Sharot, 2011; Sharot et al., 2011). Sharot et al. (2011) found that when participants re-estimated their likelihood of experiencing 80 different types of adverse life events, they updated their beliefs optimistically when they were provided with desirable information about the average probabilities of these events (i.e., when the average probability of experiencing the event was lower than the participants’ original estimate) but neutrally when new information was undesirable (i.e., when the average probability of experiencing the event was higher than the participants’ original estimate). It has also been well documented that humans are better at recollecting good events or past successes than they are at recollecting bad ones or failures (Histed et al., 2009) and are more likely to attribute past success to their own skill, while failures are attributed to bad luck (Langer & Roth, 1975; Seligman, 1990).

Despite the significant evidence on the affective incentives that motivate optimism, the asymmetric information processing and recall mechanisms that help maintain an optimistic mindset and the fact that over 1,000 articles have been published on unrealistic optimism in the last four decades (Shepperd et al., 2017), it remains untested whether this miscalibration is to some extent a consequence of cognitive (in)ability. This article attempts to address this gap in the literature.

There are reasons for expecting that the optimism bias may be associated with cognitive ability. This follows from the emphasis placed on the “two-system” or “dual-process” approach to decision-making in the heuristics and biases literature (Fudenberg & Levine, 2006; Kahneman & Frederick, 2005; Stanovich, 1999; Thaler & Shefrin, 1981). Here, the two systems are labeled as System 1, where reasoning is intuitive, effortless, impulsive, affective and emotional, and System 2, where reasoning is deliberative, logical, and analytical. This two-system model of cognition embodies the assumption that judgmental biases, like unrealistic optimism, are universal properties of System 1, a heuristic system that operates with little or no effort and no sense of voluntary control (Tversky & Kahneman, 1996). However, the presence of judgmental biases can vary from person to person because heuristic responses can sometimes be overridden by System 2, the system that allocates attention to effortful mental activities. Indeed, while System 1 arguably serves an important adaptive role in speeding up the decision-making process, there are circumstances in which a naturally primed response must be inhibited so that a deliberate and more advantageous decision can be made. Overriding a heuristically primed response may be reasonably straightforward for individuals with decreased emotional reactions from deficient emotional circuitry, as the literature attests (Shiv, Loewenstein, & Bechara, 2005; Shiv, Loewenstein, Bechara, Damasio, & Damasio, 2005). However, for individuals with stable emotional circuitry, the computational power required to override (or the ability to recognize the need to override) an inappropriate heuristic response likely depends on intelligence (Stanovich & West, 2008).

Supportive empirical evidence for this framework comes from the experimental literature on cognitive ability and judgmental biases. For instance, intelligence has been found to lower one’s susceptibility to hindsight bias (Stanovich & West, 1998), overconfidence (Bruine de Bruin et al., 2007; Stanovich & West, 1998), framing, and the sunk cost fallacy (Bruine de Bruin et al., 2007). Furthermore, studies have found that higher scores on the Cognitive Reflection Test (CRT; Frederick, 2005)—a cognitive test designed to measure people’s ability to resist reporting an incorrect “intuitive” answer and engage in further reflection to find a correct answer—are associated with smaller deviations from normative, rational-responding on a variety of tasks from the heuristics and biases literature (Benjamin et al., 2013; Frederick, 2005; Hoppe & Kusterer, 2011; Oechssler et al., 2009; Toplak et al., 2011).

In this article, we used an unbalanced panel of 36,312 respondents drawn from a nationally representative U.K. longitudinal survey and investigated whether unrealistic optimism—a bias that plays a fundamental role in behavioral science—is correlated with cognitive ability. To understand whether biased beliefs are associated with cognitive ability, it is important that beliefs are elicited in a context where they matter. That is, where there are incentives for accuracy. Importantly, while our study focuses on a very specific forecasting task, it is conducted in a context where expectations have real consequences for the respondents. Specifically, unrealistic optimism is operationalized as the difference between a person’s expectation about next year’s financial circumstances and the financial outcome that follows. Financial expectations are crucial for key household decisions such as consumption, investments and savings. For instance, according to the standard life cycle model, households who expect increases in income can increase current consumption and reduce current savings (Bachmann et al., 2015; Brown & Taylor, 2006; Springer, 1977). Importantly, research has also shown that when people make systematic forecasting errors, then this bias can impact real economic activity through its impact on these important household decisions (see for example, Caliendo & Huang, 2008; Claus & Nguyen, 2023; Sandroni & Squintani, 2013). Thus, when setting financial expectations, respondents face the trade-off between the immediate affective benefits of savoring their future successes and the downstream consequences of distorted decision-making (Brunnermeier & Parker, 2005). A further advantage is that our respondents reported financial expectations on a repeated basis, which allowed us to measure the bias with more precision, limiting issues surrounding random errors, measurement error, bad luck, and regression toward the mean. Moreover, the measure of cognitive ability used in our study covered a broad range of cognitive skills, including memory, verbal fluency, fluid reasoning and numerical reasoning.

The findings we present provide evidence that forecasting accuracy is linked to cognitive ability. Specifically, we find that higher cognitive ability is associated with a higher incidence of realism and pessimism in beliefs and a lower incidence of unrealistic optimism. Taken together, our results lead us to conclude that the rash and risky behaviors associated with excessive optimism (Shepperd et al., 2015) may be a side product of the true driver, low cognitive ability.

## Method

### Participants

To understand the correlation between optimism bias and cognitive ability, we used data from Understanding Society (USoc) 2009 to 2021 (Waves 1–12). USoc is a nationally representative annual longitudinal survey of some 40,000 households, funded by the U.K. Economic and Social Research Council. Wave 1 of USoc included 26,000 households which were recruited from Great Britain (24,800) and Northern Ireland (1,200) with a further ethnic minority booster sample included, of some 4,000 households. A further sample of households was included from the British Household Panel Survey (BHPS). The BHPS is a nationally representative annual longitudinal survey from 1991 to 2008 (Waves 1–18). As part of Wave 18, BHPS participants were asked if they would consider joining the new, larger and more wide-ranging survey Understanding Society. Almost 6,700 of just more than 8,000 BHPS participants invited to join did so. The first interviews with BHPS participants were carried out in Wave 2 of USoc.^1^ Sample attrition rates in USoc are generally low and certainly comparable to those achieved in other similar household panels. Attrition in USoc has been found to be greatest among the young, men, black people, people on lower incomes, and those residing in the Greater London area (see P. Lynn & Borkowska, 2018). USoc covers a broad range of subjects including labor market activity, household dynamics, personality, attitudes and opinions, among other things. The sample used for our empirical analysis is restricted to those who gave valid responses to the dependent, independent, and control variables used in the subsequent analyses. This yielded a final panel of 36,312 individuals with 247,234 person-Wave observations. Therefore, we observed, on average, 6.8 observations per individual.

### Measures

#### Unrealistic Optimism

An issue with a substantial proportion of the studies on unrealistic optimism is that the bias is only evaluated at the group level, that is, whether the majority of respondents’ expectations about positive (negative) events exceed (fall below) the population average. This is problematic as it is impossible to tell who is biased and who is unbiased. Of course, some people are objectively more (or less) likely to experience good (bad) events (Colvin & Block, 1994). To be able to assess the correlation between optimism bias and cognitive ability we must be able to evaluate optimism at the individual level, that is, to compute the difference between a person’s expectation and the outcome that follows. The data used in this study allowed us to follow this strict definition of optimism and also allowed us to measure optimism on a repeated basis. We measured optimism in the domain of household finances. Specifically, by the comparison of expectations about next year’s financial circumstances and the financial outcomes that followed. Household finances is an appropriate area to elicit optimism, as previous research has suggested that the bias is highest when outcomes are under the individual’s control and rely, to some extent, on the individual’s effort or ingenuity (Shepperd et al., 2013). Financial expectations were measured via responses to the following question asked in all 12 Waves of USoc:

*“Looking ahead, how do you think you will be financially a year from now, will you be. . .*

1. *Better off*
2. *Worse off than you are now*
3. *or about the same?”*

From this, we created a three-point scale of expectations, $E_{iw},$ for each individual, i, at Wave, w. The three-point scale (from −1 to +1) ranges from “worse off than you are now” to “better off.”

As previously mentioned, for optimism to be a bias, financial expectations need to be compared with financial outcomes. Financial outcomes are measured as changes in household income from one annual Wave to the next, that is, $\Delta log{\left(Y\right)}_{iw+1}=\;log{\left(Y\right)}_{iw+1}-log{\left(Y\right)}_{iw}.$ Here, Y is the self-reported current monthly income of a respondent’s household.^2^ So that our household income variable more accurately captured spending power, we deflated household income by the Consumer Price Index (2005 = 1) and by the size of the participant’s household, using the Organization for Economic Cooperation and Development’s (OECD) equivalence scale.^3^ Finally, we log transformed the variable so that changes in household income could be approximately interpreted as percentage changes.

**Figure 1.**
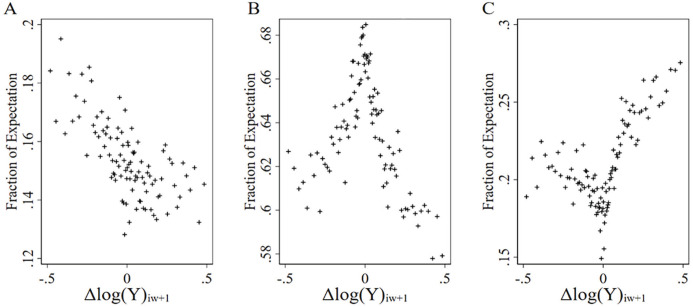
The Relationship Between Financial Expectations and Changes in Household Income. *Note.* Binned scatterplot, which plots the fraction of each financial expectation for the 100 equal-sized bins of the change in household income. For the presentation, we winsorized the change in household income distribution at the 7.5^th^ and 92.5^th^ percentiles. Sample of 36,312 individuals with 247,234 person-Wave observations. (A) Expectation: Worse off. (B) Expectation: No change. (C) Expectation: Better off.

To illustrate the degree of rationality and bias exhibited by respondents in their self-reported financial expectations, Figure 1 plots the fraction of people who reported each category of financial expectation at Wave w (of w+1 ) against changes in household income from Wave w to w+1. Specifically, we used a binned scatterplot to describe the relationship. The binned scatterplot groups the *x-axis* variable (change in household income from Wave w to w+1 ) into 100 equal-sized bins and then computes the mean of the *y-axis* variable (category of financial expectation at Wave w) within each bin, then creates a scatterplot of these data points. As depicted in Figure 1, there is substantial evidence of both rationality and bias in self-reported expectations. Specifically, in Panel A (Panel C) of Figure 1, the fraction of those who reported an expectation of “worse off” (“better off”) is increasing in the magnitude of income losses (gains). It is also the case that in Panel B of Figure 1, the fraction of those who reported an expectation of “no change” peaks when changes to household income are close to zero. However, there is also substantial evidence of bias in beliefs. For instance, the fraction who reported an expectation of “better off” but who experienced an income decrease is not zero.

The comparison of financial expectations at Wave w (of w+1) to changes in household income from Wave w to w+1, formed the basis of measuring unrealistic optimism. As financial expectations were assessed on a three-point scale, to create our measure of unrealistic optimism, we also categorized changes in household income, $\Delta log{\left(Y\right)}_{iw+1},$ on a 3-point scale (from -1 to +1) ranging from “worse off” to “better off.” This financial realization variable, $R_{iw+1},$ classified those who experienced a negative change to household income of more than 5% as “worse off.” Those who experienced a change to household income between −5% and 5% were coded as “no change” and finally, those who experienced a positive change to household income of more than 5% were classified as “better off.” The three-point financial expectation and realization scores generate nine possible combinations. Table 1 summarizes the distribution of these nine combinations. From Table 1 and consistent with the literature, respondents are found to be slightly skewed toward an optimism bias. Specifically, the difference in proportions between those making any sort of optimistic errors (35.03%, sum of three bottom left cells) compared with those making any sort of pessimistic error (33.79%, sum of three top right cells) is statistically significant, z=9.18,p<0.001. Our key dependent variable in all the analyses that follows, restricted these nine combinations to five, by constructing a 5-point scale (from −2 to +2) of unrealistic optimism, $\theta_{iw}=E_{iw}-R_{iw+1}.$ Here, 31.19% of observations are classified as “realism,” that is, the sum of the diagonal cells in Table 1 and scoring 0 on the 5-point scale. While 7.67% (5.86%) of observations are classified as “extreme optimism” (“extreme pessimism”), that is, the bottom left (top right) cell in Table 1 and scoring 2 (−2) on the five-point scale. This leaves 27.36% (27.93%) of observations classified as “moderate optimism” (“moderate pessimism”), that is, scoring 1 (−1) on the 5-point scale.

**Table 1:** Financial Expectations and Realizations (3-Point Scales).

Expectation:	Realization:			Total
	Worse off	No change	Better off	
Worse off	15,502	8,444	14,487	38,433
	(6.27%)	(3.42%)	(5.86%)	(15.55%)
No change	57,441	37,036	60,596	155,073
	(23.23%)	(14.98%)	(24.51%)	(62.72%)
Better off	18,953	10,201	24,574	53,728
	(7.67%)	(4.13%)	(9.94%)	(21.73%)
Total	91,896	55,681	99,657	
	(37.17%)	(22.52%)	(40.31%)	

*Note.* Main entries are frequencies, relative frequencies in parentheses. Sample of 36,312 individuals with 247,234 person-Wave observations.

#### Cognitive Ability

In Wave 3 of USoc, five measures of cognitive function were collected. The first measure assessed respondents’ memory, using an immediate and delayed word recall task. Specifically, participants were read a series of 10 words and immediately afterwards were asked to recall as many words as possible, in any order. Participants were then asked again, later in the interview, to recall as many words as possible. Scores from the immediate and delayed word recall task were summed together to produce a single measure, which we refer to as “Word Recall.” The second measure assessed semantic verbal fluency, where participants were given 1 min to name as many animals as possible. The final score on this item is based upon the number of unique correct responses. We refer to this measure as “Verbal Fluency.” The third measure assessed working memory, which is important for complex cognitive tasks. Here, participants were asked to give the correct answer to a series of subtraction questions. Starting at 100, the interviewer asked the respondent to subtract 7. At the next question, the respondent is asked to subtract 7 again, and so on. There is a sequence of five subtractions. The number of correct responses out of a maximum of five was recorded. We refer to this measure as “Subtraction Test.” The fourth measure assessed fluid reasoning or the ability to use abstract thought to solve novel problems. Participants were asked to write down a number sequence, as read by the interviewer. The number series consists of several numbers with a blank number in the series. The respondent is asked which number goes in the blank. Respondents were given two sets of three number sequences, where the difficulty of the second set was determined by performance in the first set. The final score is based on the correct responses from the two sets of questions while accounting for the difficulty level of the second set of problems. We refer to this measure as “Fluid Reasoning.” The last measure assessed practical numerical knowledge. Participants were asked up to five questions that were graded in complexity. Based on performance on the first 3 items, participants can get two additional (more difficult) questions or one additional (simpler) question. The types of questions asked included: “A secondhand car dealer is selling a car for £6,000. This is two thirds of what it cost new. How much did the car cost new?” and “If 5 people all have the winning numbers in the lottery and the prize is £2 million, how much will each of them get?.” The final score is based on a simple count of number of correct items. We refer to this measure as ‘Numerical Reasoning.’^4^

For the purposes of this study and the subsequent analyses, factor analysis was used to identify a general cognitive ability factor from the five cognitive function measures. This factor, known as “g,” accounted for 48% of the variance in initial eigenvalues, and all tests loaded positively on the component. In addition, the level of internal consistency across the five items appears to be high: Cronbach’s alpha is 0.72 (see Table S1 in Section A of the Supplemental Material for pairwise correlations of the cognitive function measures).

We also considered an important measurement problem. Cognitive function is highly dependent on age, with previous research having illustrated large, nonlinear variations in cognitive ability across the life course (Whitley et al., 2016). Consistently, we found that the general cognitive ability factor was subject to life cycle effects, peaking at around middle age and then falling, rather sharply, into older age. To avoid mismeasuring “g,” the effect of age was removed by regressing the general cognitive ability factor on a fourth-order polynomial of age. The predicted residuals were then used as our measure of cognitive ability in the subsequent analyses. We refer to this age-effect-free general cognitive ability factor, simply as “Cognitive Ability.” Finally, as cognitive ability is only observed in Wave 3 of USoc—while unrealistic optimism is measured repeatedly for each individual over the Waves of annual data—the cognitive ability scores were standardized and then expanded across all Waves for which the individual was observed.^5^

#### Control Variables

The focus of this study was to provide evidence of the direct relationship between unrealistic optimism and cognitive ability. In an experimental setting, the design would ideally take a group of people who vary in cognitive ability—but who are observationally equivalent in all other ways—and analyze the extent to which this variation is associated with variation in the accuracy of a forecasting task. To equalize differences across individuals we included a block of sociodemographic controls, which are thought to be in principle unaffected by cognitive ability. These control variables are age (in quadratic form); gender; marital status; the number of dependent children in the household; the square root of household size; and region of residence and Wave controls. Age and gender have been shown to be related to unrealistic optimism. For instance, there is evidence that unrealistic optimism peaks in the young and then decreases steadily into middle age (Moutsiana et al., 2013; Schwandt, 2016). Interestingly, Moutsiana et al. (2013) illustrate these life cycle effects result from the inability of the young to accurately update their beliefs in response to undesirable information. Evidence has also pointed to a lower optimism bias for females than for males (Lin & Raghubir, 2005). Marital status, the number of dependent children in the household and the square root of household size were included as household composition may affect the ability to forecast accurately. For instance, it may be easier to make realistic judgments about next year’s financial position when you live alone.

We also included the following block of variables to capture socioeconomic status: housing tenure; economic activity; logarithm of monthly household income (which is adjusted by the OECD-modified equivalence scale, deflated by the Consumer Price Index and entered in quadratic form); logarithm of monthly personal income (which is deflated by the Consumer Price Index and entered in quadratic form) and self-assessed general health. The socioeconomic controls are variables that could in principle have been affected by cognitive ability, that is, they could be interpreted as the channels behind the relationship between cognitive ability and unrealistic optimism. For instance, variables such as income, wealth, economic activity, and general health have all been linked to cognitive ability (Der et al., 2009; Meisenberg, 2012; Smith et al., 2010). While higher socioeconomic status, in turn, has been linked with both dispositional optimism—generalized outcome expectancies that good things will happen—and positive illusions (Boehm et al., 2015; Heinonen et al., 2006; S. E. Taylor & Brown, 1988).

Finally, we controlled for educational attainment. While cognitive ability has been identified as the major source of variation in educational attainment (R. Lynn & Mikk, 2009; Ritchie & Tucker-Drob, 2018; Strenze, 2007), education is potentially an important control as it may be related to greater exposure to more normative models of human judgment (Stanovich & West, 1998).

Table S2 in Section A of the Supplemental Material presents an overview of the sample characteristics, and Table 2 shows the correlations between a selection of the key variables used in the study. From Table S2, the mean age of the sample is approximately 51 years. Just more than 56% of the sample is female and 28.0% reported having a university or college degree. Just under 58% of the sample reported being married, 76.7% reported owning a house (with and without a mortgage), and finally, just more than 52% of the sample reported being in paid employment.

**Table 2:** Pairwise Correlations of Unrealistic Optimism, Cognitive Ability and Control Variables.

Variables	(1)	(2)	(3)	(4)	(5)	(6)	(7)	(8)	(9)	(10)
1. Extreme pessimism										
2. Moderate pessimism	–.155***									
3. Realism	–.168***	–.419***								
4. Moderate optimism	–.153***	–.382***	–.413***							
5. Extreme optimism	–.072***	–.179***	–.194***	–.177***						
6. Cognitive ability	–.003	–.014***	.032***	–.008***	–.015***					
7. Age (years)	.054***	.099***	–.036***	.018***	–.181***	.031***				
8. Male	–.005**	–.014***	.012***	–.006***	.017***	.130***	.034***			
9. Household income	–.109***	–.161***	–.004*	.165***	.097***	.257***	–.098***	.059***		
10. General health	–.066***	–.033***	.019***	.018***	.049***	.164***	–.213***	.009***	.199***	
11. Educational attainment	–.022***	–.043***	.026***	–.004**	.054***	.379***	–.261***	.034***	.349***	.228***

*Note.* Sample of 36,312 individuals with 247,234 person-Wave observations. The 5-point unrealistic optimism scale is included as five binary indicators for each category. Cognitive ability is our standardized age-effect-free general cognitive ability factor. Household income is the logarithm of monthly household income which has been adjusted by the Organization for Economic Co-operation and Development-modified equivalence scale and deflated by the Consumer Price Index. General health is included as a 5-point scale from 1 = *Poor* to 5 = *Excellent*. Educational attainment is included as a 6-point scale from 1 = *No formal qualifications* to 6 = *University/college degree*.

* *p* < .05. **p < .01. ***p < .001.

From Table 2, cognitive ability is positively correlated with socioeconomic factors such as education, income, and health. These socioeconomic factors—household income in particular—are in turn positively (negatively) correlated with excessively optimistic (pessimistic) beliefs.^6^ Counter to this potentially important channel through which cognitive ability may be associated with unrealistic optimism, Table 2 finds a negative correlation between cognitive ability and excessive optimism. We also find a negative (positive) correlation between cognitive ability and pessimistic (realistic) beliefs, although the correlation with “extreme pessimism” is close to zero and not statistically significant.

The data that support the findings of this study are publicly available from the U.K. Data Archive. The codebook and complete STATA analysis script to replicate the results is openly available in Open Science Framework (OSF) at: https://osf.io/aqf2m/?view_only=dbb9d8542a0d4da799b36a466576caac.

### Analytic Strategy

Most studies on unrealistic optimism only have a single cross-sectional component, however, here we had the rare chance to measure unrealistic optimism on a repeated basis for a common “real-world” task. This is advantageous, as unrealistic optimism, as measured in a certain Wave, is likely to comprise of substantial measurement error. Specifically, unrealistic optimism measured in one Wave will capture both the systematic psychological bias associated with biased expectations and a stochastic component, capturing random errors of evaluation and unforeseeable shocks to household income. In this view, to minimize the stochastic component, we assessed a pooled sample (i.e., combining observations from multiple Waves into a single sample) which allowed estimation of the relationship between cognitive ability and repeated individual measures of unrealistic optimism—an average of 6.8 observations per person. As our dependent variable, $\theta_{iw}$ has five possible outcomes j=(−2,−1,0,1,2), we estimated the following equation via a multinomial logistic regression, for the i *i*th individual at Wave w:

$$\begin{array}{l} \theta_{iwj}^{*}=\beta_{j}CognitiveAbility_{i}+\alpha_{j}Controls_{iw}\\ \;\;\;\;\;\;\;\;\;\;+\epsilon_{iwj},\;(i=1,\;\ldots .,N;w=1,\ldots .,W_{i}) \end{array}$$

where $\theta_{iwj}^{*}$ is the individual’s latent probability of choosing outcome j, in each Wave of the sequence $W_{i}.$ *CognitiveAbility_i_* is our standardized age-effect-free general cognitive ability factor and $\beta_{j}$ are the coefficients of interest, estimated from between-person variation. $Controls_{iw}$ represents the control variables described above and finally, $\epsilon_{iwj}$ which accounts for the unobserved randomness. Multinomial logistic regression is a simple extension of binary logistic regression, but which allows the researcher to estimate the probability of observing an outcome for a dependent variable that has more than two categories. Importantly, it also allows for independent variables, such as cognitive ability, to have different effects on the probability of observing each outcome (i.e., *$\beta_{j}).$* This is in contrast to ordered logistic regression—which relies on the proportional odds or parallel regression assumption—where no independent variable can have a disproportionate effect on a specific level of the dependent variable. As the research question is about how a change in a continuous independent variable affects the probability of observing an outcome, we follow the recommended practice of presenting the results in terms of average marginal effects (AME’s; Norton & Dowd, 2018; Williams, 2012).

We consider the AMEs across nested models. In Model 1, we estimated the relationship between cognitive ability and unrealistic optimism with the blocks of sociodemographic and socioeconomic controls. In Model 2, we add controls for educational attainment. These models, by including covariates that could in principle have been affected by cognitive ability—that is, blocking the potentially important indirect paths between cognitive ability and unrealistic optimism identified in Table 2—allowed us to isolate the direct relationship between cognitive ability and miscalibrated beliefs (Cinelli et al., 2024).

While this is our preferred analytic strategy, Section B of the Supplemental Material presents alternative methods. All procedures yield similar conclusions.

## Results

**Figure 2.**
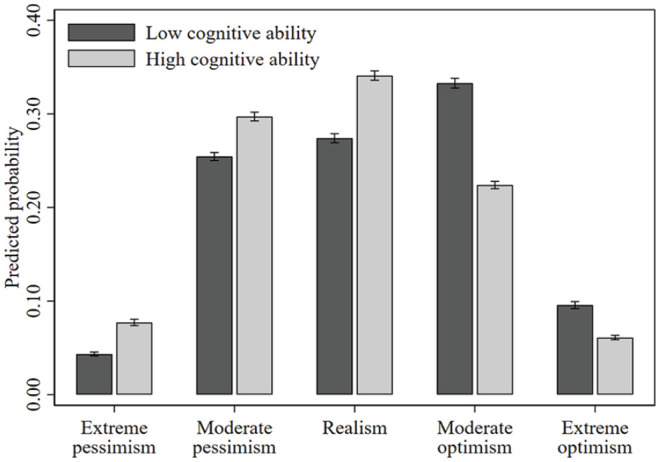
The Predicted Relationship Between Cognitive Ability and the 5-Point Unrealistic Optimism Scale—Sociodemographic and Socioeconomic Controls Included (Model 1, Table 3). *Note.* Bars (with 95% confidence intervals) represent the predicted probabilities of unrealistic optimism, for those high (+2 standard deviations from the mean) and low (−2 standard deviations from the mean) on cognitive ability.

**Figure 3:**
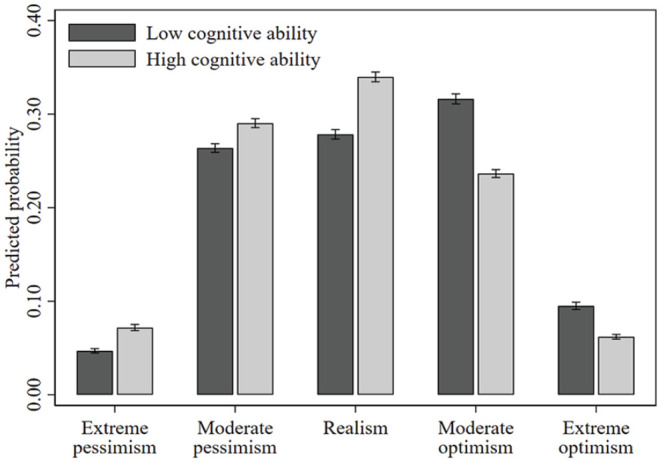
The Predicted Relationship Between Cognitive Ability and the 5-Point Unrealistic Optimism Scale—Sociodemographic, Socioeconomic and Educational Attainment Controls Included (Model 2, Table 3). *Note.* Bars (with 95% confidence intervals) represent the predicted probabilities of unrealistic optimism, for those high (+2 standard deviations from the mean) and low (−2 standard deviations from the mean) on cognitive ability.

Table 3 reports the main findings. As noted above, for ease of exposition and following convention, we report the AME of a one standard deviation increase in our age-effect-free general cognitive ability factor on the probability of observing each of the five outcomes of our dependent variable. An alternative representation of the results in Table 3 is provided in Figures 2 and 3, were we plotted the average predicted probability of observing each of the five outcomes for those low (−2 standard deviations from the mean) and high (+2 standard deviations from the mean) on cognitive ability.

**Table 3.** Multinomial Logistic Regressions Measuring the Relationship Between Cognitive Ability and the 5-Point Unrealistic Optimism Scale.

	Dependent variable: 5-point unrealistic optimism scale				
Predictors	Extreme pessimism	Pessimism	Realism	Optimism	Extreme optimism
Model 1: Sociodemographic and socioeconomic controls					
Cognitive ability	0.008*** [0.007, 0.010]	0.011*** [0.009, 0.013]	0.017*** [0.015, 0.019]	–0.027*** [–0.029, –0.025]	–0.009*** [–0.010, –0.007]
Model 2: Sociodemographic, socioeconomic and educational attainment controls					
Cognitive ability	0.006*** [0.005, 0.008]	0.007*** [0.004, 0.009]	0.015*** [0.013, 0.018]	–0.020*** [–0.022, –0.018]	–0.008*** [–0.010, –0.007]
AME Model 2—AME Model 1	{–0.002}***	{–0.004}***	{–0.001}***	{0.007}***	{0.001}*
Observations	247,234				
Individuals	36,312				

*Note.* Main entries are AMEs, 95% confidence intervals using clustered standard errors by the individual in brackets. In curly brackets we report tests for the difference in AMEs across the models, following the procedure in Mize et al. (2019). Cognitive ability is our standardized age-effect-free general cognitive ability factor. Sociodemographic controls include age (in quadratic form); gender; marital status; the number of dependent children in the household; the square root of household size; and region of residence and Wave controls. Socioeconomic controls include housing tenure; economic activity; logarithm of monthly household income (which is adjusted by the Organization for Economic Co-operation and Development-modified equivalence scale, deflated by the Consumer Price Index and entered in quadratic form); logarithm of monthly personal income (which is deflated by the Consumer Price Index and entered in quadratic form) and self-assessed general health. Educational attainment controls represent the highest level of attainment. For full results of Model 2 see Table S3 in Section A of the Supplemental Material. The relative frequency for each category of our dependent variable is as follows: “Extreme pessimism” = 0.06; “Moderate pessimism” = 0.28; “Realism” = 0.31; “Moderate optimism” = 0.27; “Extreme optimism” = 0.08. AME = Average marginal effect.

* *p* < .05. **p < .01. ***p < .001.

We begin by discussing the estimated relationship between cognitive ability and unrealistic optimism while controlling for sociodemographic and socioeconomic factors, as reported in Model 1 of Table 3 (Figure 2). For instance, a one standard deviation increase in cognitive ability, increases the probability of being in the “extreme optimism” (“moderate optimism”) category by 0.9 (2.7) percentage points. Alternatively, from Figure 2, those low (-2 standard deviations from the mean) on cognitive ability have a predicted probability of “extreme optimism” (“moderate optimism”) of 9.6% (33.3%), while those high (+2 standard deviations from the mean) on cognitive ability have a probability of 6.1% (22.4%), a relative decrease of 3.5 (10.9) percentage points or 36.3% (32.7%). In the same way, relative to those low on cognitive ability, those high on cognitive ability have a 78.2%, 16.8%, and 24.4% increased probability of “extreme pessimism,” “moderate pessimism,” and “realism,” respectively. Indeed, Figure 2, reports a distribution of beliefs skewed toward excessive optimism for those low on cognitive ability and skewed toward both realistic and pessimistic beliefs for those high on cognitive ability.

In Model 2 of Table 3 (Figure 3), we included controls for educational attainment. As cognitive ability has been identified as the major source of variation in educational attainment (R. Lynn & Mikk, 2009), these collinear variables may contain the same information about the dependent variable. However, educational attainment could be interpreted as a further channel behind the relationship between cognitive ability and unrealistic optimism, if education is related to greater exposure to more normative models of human judgment (Stanovich & West, 1998). We report in Table 3, following Mize et al. (2019), tests for the difference in AME’s between Model 1 and Model 2. Adding educational attainment did not affect the pattern of results; however, its inclusion significantly reduced the effect sizes. From Figure 3, those low on cognitive ability have a predicted probability of “extreme optimism” (“moderate optimism”) of 9.5% (31.6%), while the respective estimate for those high on cognitive ability is 6.2% (23.6%), a relative decrease of 34.8% (25.2%). In the same way, relative to those low on cognitive ability, those high on cognitive ability have a 53.2%, 10.1% and 22% increased probability of “extreme pessimism,” ‘moderate pessimism’ and “realism,” respectively.

Finally, while we reported the AME of cognitive ability on unrealistic optimism, averages can sometimes obscure differences in effects across cases. Indeed, in nonlinear models, such as multinomial logit, the AME is not constant, and it varies not only by cognitive ability, but also across the characteristics of the individual. Specifically, from Figure 3, while those low on cognitive ability have on average an increased probability of “extreme optimism” of 3.3 percentage points compared to those high on cognitive ability, at the individual level the largest effect was an increased probability of 11.9 percentage points. These larger individual effects tended to be among the young, where unrealistic optimism is most prevalent (Moutsiana et al., 2013; Schwandt, 2016).

One potential issue in our analysis arises from the somewhat arbitrary categorization of financial realizations. For instance, it is not obvious that people considered changes to household income of between -5% and 5%, when they reported a financial expectation of “no change.” For this reason, we repeated the main analysis, but where the three-point financial realizations scale was coded around a “no change” in household income from Wave w to w+1 of between −2.5% and 2.5%, −7.5% and 7.5% and finally, −12.5% and 12.5%. Here we found, regardless of how we categorize financial realizations, the main results continue to hold (see Tables S4–S6 in Section A of the Supplemental Material).

There is also the related limitation regarding the lack of an exact parallel between the (subjective) financial expectation question and the (objective) financial realization measure. Specifically, financial realizations are measured from changes in household income, while the financial expectation question is about future financial well-being in general. While it does seem intuitive to think that when people are asked about their financial expectations, they would view themselves as part of the household in which they reside, people may view this question more narrowly. For this reason, we repeated the analysis but where financial realizations were constructed from changes in personal income. The results are highly robust to this alternate approach (see Table S7 in Section A of the Supplemental Material).

Finally, as we analyzed an unbalanced panel, individuals differed in the number of Waves in which we observed their level of unrealistic optimism. This means that for some individuals we measured their unrealistic optimism with less precision. To mitigate these concerns, we repeated the main analysis, but restricting our sample to only those individuals who were observed in at least 7 Waves. This leaves a sample of 20,266 individuals with 191,176 person-Wave observations. While this procedure may have introduced some survivorship bias, the results are wholly consistent with those reported in the main text (see Table S8 in Section A of the Supplemental Material).

## General Discussion

It is a puzzle why humans tend toward unrealistic optimism when normative models of human judgment, like expected utility theory, suggest unbiased assessments of probabilities are advantageous. In answering this puzzle, the literature has focused on the affective benefits of unrealistic optimism (S. E. Taylor & Brown, 1988), despite recent evidence which has suggested it may be realists that do best on the well-being front (de Meza & Dawson, 2021). Here, we provide evidence from a nationally representative longitudinal survey, that unrealistic optimism, one of the most established psychological “biases” that plays a crucial role in behavioral science, is associated with cognitive ability. We do this in the context of financial expectations, which are fundamental to key household decisions such as saving, investment and consumption. Therefore, the accuracy of elicited expectations has “real-world” consequences for participants. The effects are not small, with those highest on cognitive ability experiencing a 22% (53.2%) increase in the probability of “realism” (“extreme pessimism”) and a 34.8% decrease in the probability of “extreme optimism” relative to those lowest on cognitive ability.

Forecasting the future with accuracy is difficult, and for this reason alone, errors in forecasting, both optimistic and pessimistic, may be more likely to arise for those low on cognitive ability. However, all else being equal, our results do not indicate that low cognitive ability leads to an increased probability of both self-deprecating and self-flattering biases (Coelho, 2010), just self-flattering biases. This pattern of results is though consistent with the idea that intelligence governs the ease to which the autonomous System 1 response can be overridden (or indeed, the ability to recognize the need to override). The mechanism through which unrealistic optimism—perhaps a universal part of our naturally primed heuristic response system—is overridden by intelligence is an open question. For instance, are those high on cognitive ability more able to resist the immediate affective benefits of unrealistic optimism and recognize the need for more realistic or cautious expectations, which may lead to present gloom but also to better decision-making? Or are those high on cognitive ability more symmetric in how they incorporate (or attribute) undesirable and desirable information, from their past or present, into their current beliefs? Indeed, the optimism bias can only be maintained if individuals update their beliefs optimistically when new information is positive but neutrally when new information is negative (Drobner, 2022; Eil & Rao, 2011; Sharot, 2011; Sharot et al., 2011) or, in a similar way, if people attribute past success to their own skill and failure to bad luck (Langer & Roth, 1975; Seligman, 1990). These seem like fruitful areas for future research.

Alongside the large body of literature on unrealistic optimism, sits the literature on overconfidence—overestimation or excessive precision in one’s estimates of own ability (Moore & Healy, 2008). As with unrealistic optimism, overconfidence has been found to be a pervasive human trait with important affective benefits, such as maintaining self-esteem (Dunning et al., 1995; Köszegi, 2006; Kunda, 1987) while also being linked to a catalog of decision errors resulting in substantial personal costs. These have included excessive trading; undertaking value-destroying mergers; risky driving behavior; entrepreneurial failures; and problem gambling (Barber & Odean, 2001; Camerer & Lovallo, 1999; Davis et al., 2000; Koellinger et al., 2007; Malmendier & Tate, 2008; Schlehofer et al., 2010). Within this literature, which is now famously known as the Dunning-Kruger effect, Kruger and Dunning (1999) illustrated that poor performers in a certain domain are the most likely to overestimate their ability in that domain, whereas top performers make more accurate self-assessments. While the current study goes beyond the discrepancy between objective and subjective ability in a particular domain, our findings could be explained by the metacognitive account of the Dunning-Kruger effect. Specifically, people low on cognitive ability may not only perform tasks related to future income poorly but also lack the metacognitive ability to realize their own incompetence (Dunning et al., 2003; Kruger & Dunning, 1999).

A handful of articles have investigated the link between cognitive ability and the alternate psychological perspective of optimism, dispositional optimism. Here, the evidence suggests that looking on the bright side of life is a hallmark of high cognition (Karhu et al., 2022; Lounsbury et al., 2005; Nurmi & Pulliainen, 1991; A. M. Taylor et al., 2017). How do we reconcile these results with our claim that those high in cognitive ability tend to look more on the right side? In addressing this, we apply the logic of de Meza and Dawson (2021), who suggest the well-established relationship between dispositional optimism and psychological well-being (Scheier & Carver, 1987) may partly reflect the realistic expectation of people likely to have positive experiences. In this view, the positive relationship between intelligence and dispositional optimism could simply be that those high on cognitive ability have more to be optimistic about. This problem is eliminated by examining the relationship between expectations and cognitive ability while controlling for outcomes, as we employed in the current study.

Despite the inclusion of many control variables, the negative correlation between cognitive ability and unrealistic optimism could, as always, be explained by omitted variable bias. For instance, it is possible that those low on cognitive ability may self-select into environments that make financial forecasting more difficult. However, the implication is that those low on cognitive ability will make more optimistic and pessimistic errors than those high on cognitive ability, which we do not find. In addition, it is possible that intelligence may be correlated with some aspect of personality that is incompatible with optimistic beliefs. Indeed, evidence points to relations between intelligence and broad personality factors (e.g., Anglim et al., 2022; Stanek & Ones, 2023) and between broad personality factors and dispositional optimism (Sharpe et al., 2011). In Wave 3 of USoc, we had available the short 15-item Big-Five inventory (BFI-15). However, including personality factors—Openness, Neuroticism, Extraversion, Conscientiousness and Agreeableness—in our analysis had almost no effect on the estimated relationship between cognitive ability and optimism bias (see Tables S9 and S10 in Section A of the Supplemental Material).

A further consideration is the limitation that our unrealistic optimism variable is not particularly fine-grained. For instance, financial expectations are measured on a 3-point scale, ranging from “worse off than you are now” to “better off.” In experimental settings, researchers could ask the alternative, “*Please indicate how much you think your income will change by this time next year*.” While this approach would arguably yield more variance in optimism bias between individuals, experimental samples are not typically nationally representative—most studies are conducted on college undergraduates, who are widely perceived as reasonably homogeneous—or reinterviewed over a decade. Which would yield concerns over external validity and measurement error.

Despite these limitations, our results lead us to make some final important conclusions. Unrealistically optimistic financial expectations can lead to excessive levels of current consumption and debt, as well as insufficient savings (Caliendo & Huang, 2008; Claus & Nguyen, 2023; Sandroni & Squintani, 2013). It can also lead to excessive business entries and subsequent failures, as optimists overestimate the financial returns from entrepreneurship (de Meza et al., 2019). Our findings suggest that these supposed consequences of optimism bias, may be a side product of the true driver, low cognitive ability. In a similar way, unrealistic optimism may mediate the important relationship between cognitive ability and financial vulnerability (Gladstone & Barrett, 2023). While unrealistic optimism about household finances is a very specific measure of optimism, to the extent to which unrealistic optimism is domain-general, cognitive ability could also explain why optimists fail to take precautionary actions outside the realm of finance, like quitting smoking (Dillard et al., 2006). Finally, it would seem natural to conclude that unrealistic optimism will be unimportant in markets where participants are likely to be high on cognitive ability (Oechssler et al., 2009). However, even those high on cognitive ability display significant errors in judgment.

## Supplemental Material

sj-docx-1-psp-10.1177_01461672231209400 – Supplemental material for Looking on the (B)right Side of Life: Cognitive Ability and Miscalibrated Financial Expectations Supplemental material, sj-docx-1-psp-10.1177_01461672231209400 for Looking on the (B)right Side of Life: Cognitive Ability and Miscalibrated Financial Expectations by Chris Dawson in Personality and Social Psychology Bulletin

## Notes

1. A further sample of households called the Immigrant and Ethnic Minority Boost Sample (IEMBS), was added in Wave 6. This was approximately 2,900 households selected from areas of high ethnic minority concentration where at least one member was born outside the United Kingdom, or from an ethnic minority group. This sample was not included in our analysis as these households were recruited after the collection of cognitive function measures, which occurred at Wave 3.

2. Household income is a derived variable and can be decomposed into six self-reported subcomponents: labor income; miscellaneous income; private benefit income; investment income; pension income; and social benefit income.

3. The OECD equivalence scale is given by 1 + (number of adults – 1) × 0.6 + number of children × 0.4.

4. McFall (2013) provides further details of the data collection methods and procedures used to measure cognitive function in USoc.

5. Residualizing “g” on age, while important, has little impact on the estimated coefficients in the subsequent analyses.

6. Why socioeconomic status, and income in particular, matters for unrealistic optimism is open to debate. Perhaps those low in income are more exposed to financial threats and therefore may use negative information more prominently when formulating financial expectations. This is consistent with Garrett et al. (2018) who illustrate that asymmetric information integration, one of the mechanisms thought to generate and sustain the optimism bias, evaporates under threat.
