# Supplementary material for: Looking on the (B)right Side of Life: Cognitive Ability and Miscalibrated Financial Expectations
Source: Pers Soc Psychol Bull. 2023 Nov 10;51(9):1703–15. doi: 10.1177/01461672231209400 (PMC12276405; doi:10.1177/01461672231209400)
Supplement: sj-docx-1-psp-10.1177_01461672231209400 – Supplemental material for Looking on the (B)right Side of Life: Cognitive Ability and Miscalibrated Financial Expectations [file sj-docx-1-psp-10.1177_01461672231209400.docx]

**Supplemental Material**

**For**

**“Looking on the (b)right side of life: Cognitive ability and miscalibrated financial expectations”**

**Section A**

Tables S1, S2, S3, S4, S5, S6, S7, S8, S9 and S10

**Section B**

Tables S11, S12 and S13

**References**

Mize, T. D., Doan, L., & Long, J. S. (2019). A general framework for comparing predictions and marginal effects across models. *Sociological Methodology*, *49*(1), 152-189.

**Section A**

**Table S1.** Pairwise correlations of cognitive function measures

| Variables | (1) | (2) | (3) | (4) |
| --- | --- | --- | --- | --- |
| (1) Word recall |  |  |  |  |
| (2) Verbal fluency | .377*** |  |  |  |
| (3) Subtraction test | .186*** | .192*** |  |  |
| (4) Fluid reasoning | .333*** | .317*** | .366*** |  |
| (5) Numerical reasoning | .290*** | .314*** | .378*** | .496*** |

*Note.* Sample of 36,312 individuals. The average interitem correlation for the five cognitive function measures is 0.34.

* p<0.05, ** p<0.01, *** p<0.001.

**Table S2.** Summary statistics

|  | *Mean/*  *Frequency* | *Std. Dev.* |
| --- | --- | --- |
| **5-point unrealistic optimism scale:** |  |  |
| Extreme pessimism | 0.0586 |  |
| Moderate pessimism | 0.279 |  |
| Realism | 0.312 |  |
| Moderate optimism | 0.274 |  |
| Extreme optimism | 0.0767 |  |
| **Cognitive function measures:** |  |  |
| Word recall | 11.96 | 3.232 |
| Verbal fluency | 22.69 | 6.505 |
| Subtraction test | 4.537 | 0.927 |
| Fluid reasoning | 533.1 | 29.51 |
| Numerical reasoning | 3.771 | 0.993 |
| **Sociodemographic control variables:** |  |  |
| Age (years) | 50.90 | 16.75 |
| Male | 0.439 |  |
| Married | 0.577 |  |
| Living as couple | 0.108 |  |
| Widowed, divorced or separated | 0.154 |  |
| Single, never married | 0.161 |  |
| Number of children in household | 0.537 | 0.925 |
| Square root of household size | 1.595 | 0.399 |
| **Socioeconomic control variables:** |  |  |
| Own house outright | 0.369 |  |
| Own house with mortgage | 0.398 |  |
| Local authority renter | 0.130 |  |
| Private sector renter | 0.102 |  |
| Employee | 0.523 |  |
| Self-employed | 0.0791 |  |
| Unemployed | 0.0297 |  |
| Full-time education | 0.0227 |  |
| Retired | 0.266 |  |
| Economically inactive | 0.0799 |  |
| Logarithm of monthly household income | 7.519 | 0.634 |
| Logarithm of monthly personal income | 7.238 | 0.987 |
| General health: Excellent | 0.147 |  |
| General health: Very good | 0.354 |  |
| General health: Good | 0.306 |  |
| General health: Fair | 0.143 |  |
| General health: Poor | 0.0497 |  |
| **Educational attainment:** |  |  |
| University/College degree | 0.280 |  |
| Other higher degree | 0.133 |  |
| A-Level | 0.201 |  |
| GCSE | 0.199 |  |
| Other qualification | 0.0914 |  |
| No qualification | 0.0954 |  |
| **15-item Big-Five inventory (BFI-15):** |  |  |
| Openness | 4.570 | 1.241 |
| Neuroticism | 3.540 | 1.389 |
| Extraversion | 4.584 | 1.266 |
| Conscientious | 5.511 | 1.037 |
| Agreeableness | 5.638 | 0.976 |
| Observations | 247,234 |  |
| Individuals | 36,312 |  |

*Note.* The logarithm of monthly household income is adjusted by the OECD-modified equivalence scale and deflated by the Consumer Price Index. The logarithm of monthly personal income is deflated by the Consumer Price Index. Education attainment is measured as a set of binary variables indicating the highest level of attainment. These binary variables are: university or college degree - either at undergraduate or postgraduate level; other higher degree - which includes diplomas in higher education, teaching qualifications, nursing or other medical qualifications; A-level – which are post-compulsory examinations taken at 18 to qualify for college or university entrance; GCSE – which are schooling attainment qualifications taken at 16; other qualification – which includes school leaving exam certificate or matriculation; and lastly, no formal qualifications. Personality traits—Openness, Neuroticism, Extraversion, Conscientiousness and Agreeableness—are measured using the short 15-item Big-Five inventory (BFI-15). Each trait is based on a level of agreement with three statements, assessed on a seven-point scale. Responses are added across each set of the three statements and then divided by the number of items over which the sum is calculated.

**Table S3.** Multinomial logistic regression measuring the relationship between cognitive ability and the 5-point unrealistic optimism scale

|  | Dependent variable: 5-point unrealistic optimism scale | | | | |
| --- | --- | --- | --- | --- | --- |
|  | Extreme pessimism | Pessimism | Realism | Optimism | Extreme optimism |
| Cognitive ability | 0.006*** | 0.007*** | 0.015*** | -0.020*** | -0.008*** |
|  | [0.005, 0.008] | [0.004, 0.009] | [0.013, 0.018] | [-0.022, -0.018] | [-0.010, -0.007] |
| Age (years) | 0.001*** | 0.002*** | -0.002*** | 0.001*** | -0.002*** |
|  | [0.000, 0.001] | [0.002, 0.002] | [-0.002, -0.002] | [0.001, 0.002] | [-0.003, -0.002] |
| Male | -0.003* | -0.011*** | 0.011*** | -0.006** | 0.009*** |
|  | [-0.005, -0.000] | [-0.015, -0.007] | [0.006, 0.015] | [-0.010, -0.002] | [0.007, 0.012] |
| Married | 0.002 | 0.024*** | 0.011** | -0.025*** | -0.013*** |
|  | [-0.002, 0.006] | [0.017, 0.031] | [0.004, 0.018] | [-0.032, -0.017] | [-0.017, -0.008] |
| Living as couple | -0.003 | 0.016*** | 0.014*** | -0.024*** | -0.004 |
|  | [-0.008, 0.001] | [0.008, 0.024] | [0.006, 0.023] | [-0.031, -0.016] | [-0.008, 0.001] |
| Widowed, divorced or separated | 0.004 | 0.001 | -0.009* | -0.006 | 0.010** |
|  | [-0.000, 0.008] | [-0.006, 0.009] | [-0.017, -0.001] | [-0.015, 0.002] | [0.004, 0.016] |
| Number of children in household | -0.014*** | -0.032*** | 0.007*** | 0.033*** | 0.006*** |
|  | [-0.016, -0.012] | [-0.035, -0.029] | [0.004, 0.010] | [0.030, 0.036] | [0.005, 0.008] |
| Square root of household size | 0.032*** | 0.049*** | -0.075*** | -0.014*** | 0.009*** |
|  | [0.027, 0.036] | [0.041, 0.056] | [-0.083, -0.067] | [-0.022, -0.006] | [0.004, 0.013] |
| Own house outright | 0.012*** | 0.031*** | -0.001 | 0.001 | -0.042*** |
|  | [0.008, 0.016] | [0.024, 0.038] | [-0.009, 0.007] | [-0.007, 0.008] | [-0.047, -0.037] |
| Own house with mortgage | 0.010*** | 0.044*** | 0.008* | -0.030*** | -0.032*** |
|  | [0.006, 0.014] | [0.037, 0.051] | [0.001, 0.015] | [-0.036, -0.023] | [-0.036, -0.028] |
| Local authority renter | 0.005* | -0.000 | -0.020*** | 0.015*** | 0.001 |
|  | [0.001, 0.009] | [-0.008, 0.007] | [-0.029, -0.012] | [0.007, 0.023] | [-0.005, 0.007] |
| Self-employed | -0.013*** | -0.023*** | -0.052*** | 0.027*** | 0.060*** |
|  | [-0.017, -0.009] | [-0.030, -0.015] | [-0.059, -0.045] | [0.020, 0.035] | [0.055, 0.066] |
| Unemployed | -0.003 | -0.103*** | 0.043*** | 0.020** | 0.044*** |
|  | [-0.009, 0.002] | [-0.113, -0.094] | [0.030, 0.055] | [0.007, 0.033] | [0.035, 0.052] |
| Full-time education | 0.029*** | -0.005 | -0.034*** | 0.018* | -0.009** |
|  | [0.019, 0.040] | [-0.019, 0.010] | [-0.047, -0.020] | [0.004, 0.033] | [-0.015, -0.004] |
| Retired | -0.003 | -0.028*** | 0.024*** | 0.013*** | -0.006 |
|  | [-0.007, 0.001] | [-0.035, -0.021] | [0.016, 0.033] | [0.005, 0.021] | [-0.012, 0.000] |
| Economically inactive | 0.005* | -0.027*** | -0.024*** | 0.039*** | 0.007** |
|  | [0.001, 0.009] | [-0.035, -0.020] | [-0.032, -0.016] | [0.031, 0.047] | [0.002, 0.012] |
| Logarithm of monthly household income | -0.052*** | -0.173*** | -0.025*** | 0.192*** | 0.059*** |
|  | [-0.055, -0.050] | [-0.178, -0.168] | [-0.030, -0.020] | [0.187, 0.197] | [0.056, 0.062] |
| Logarithm of monthly personal income | 0.003** | 0.003 | -0.003 | -0.004* | 0.001 |
|  | [0.001, 0.005] | [-0.000, 0.007] | [-0.007, 0.001] | [-0.008, -0.001] | [-0.002, 0.003] |
| General health: Excellent | -0.044*** | 0.003 | 0.011* | 0.022*** | 0.008* |
|  | [-0.050, -0.038] | [-0.006, 0.013] | [0.000, 0.021] | [0.012, 0.032] | [0.002, 0.015] |
| General health: Very good | -0.042*** | 0.000 | 0.010* | 0.025*** | 0.006 |
|  | [-0.048, -0.036] | [-0.008, 0.009] | [0.001, 0.020] | [0.016, 0.034] | [-0.000, 0.013] |
| General health: Good | -0.035*** | -0.004 | 0.005 | 0.025*** | 0.009** |
|  | [-0.041, -0.029] | [-0.012, 0.005] | [-0.005, 0.015] | [0.016, 0.034] | [0.002, 0.015] |
| General health: Fair | -0.023*** | -0.009 | 0.000 | 0.025*** | 0.006 |
|  | [-0.029, -0.017] | [-0.018, 0.000] | [-0.010, 0.010] | [0.015, 0.034] | [-0.001, 0.013] |
| University/College degree | 0.024*** | 0.042*** | 0.011* | -0.082*** | 0.006 |
|  | [0.019, 0.028] | [0.034, 0.049] | [0.002, 0.021] | [-0.091, -0.073] | [-0.001, 0.012] |
| Other higher degree | 0.007** | 0.022*** | 0.004 | -0.051*** | 0.018*** |
|  | [0.003, 0.012] | [0.014, 0.030] | [-0.005, 0.014] | [-0.060, -0.042] | [0.011, 0.024] |
| A-Level | 0.005* | 0.006 | 0.006 | -0.034*** | 0.017*** |
|  | [0.001, 0.009] | [-0.001, 0.013] | [-0.003, 0.015] | [-0.043, -0.026] | [0.011, 0.023] |
| GCSE | 0.003 | 0.009* | -0.003 | -0.025*** | 0.016*** |
|  | [-0.001, 0.007] | [0.002, 0.016] | [-0.012, 0.006] | [-0.034, -0.017] | [0.010, 0.022] |
| Other qualification | 0.001 | 0.003 | 0.001 | -0.019*** | 0.014*** |
|  | [-0.004, 0.005] | [-0.005, 0.010] | [-0.009, 0.011] | [-0.028, -0.009] | [0.007, 0.021] |
| Observations | 247,234 |  |  |  |  |
| Individuals | 36,312 |  |  |  |  |

*Note.* Main entries are AME’s, 95% confidence intervals using clustered standard errors by individual in brackets. Cognitive ability is our standardized age-effect-free general cognitive ability factor. The logarithm of monthly household income is adjusted by the OECD-modified equivalence scale and deflated by the Consumer Price Index. The logarithm of monthly personal income is deflated by the Consumer Price Index. Age and the income variables are all entered into the regression in quadratic form, we report the AME of the component terms. Regression includes region of residence and Wave controls. The relative frequency for each category of our dependent variable is as follows: ‘Extreme pessimism’ = 0.06; ‘Moderate pessimism’ = 0.28; ‘Realism’ = 0.31; ‘Moderate optimism’ = 0.27; ‘Extreme optimism’ = 0.08. AME = Average marginal effect.

* p<0.05, ** p<0.01, *** p<0.001.

**Table S4.** Multinomial logistic regressions measuring the relationship between cognitive ability and the 5-point unrealistic optimism scale. Financial realizations coded around a ‘no change’ in household income of between -2.5% and 2.5%.

|  | Dependent variable: 5-point unrealistic optimism scale | | | | | |
| --- | --- | --- | --- | --- | --- | --- |
|  | Extreme pessimism | Pessimism | Realism | Optimism | Extreme optimism |  |
| Model 1: Sociodemographic and socioeconomic controls | | | | | |  |
| Cognitive ability | 0.010*** | 0.010*** | 0.017*** | -0.028*** | -0.008*** |  |
|  | [0.009, 0.011] | [0.007, 0.012] | [0.015, 0.019] | [-0.030, -0.026] | [-0.010, -0.007] |  |
| Model 2: Sociodemographic, socioeconomic and educational attainment controls | | | | | |  |
| Cognitive ability | 0.008*** | 0.006*** | 0.014*** | -0.020*** | -0.008*** |  |
|  | [0.006, 0.009] | [0.004, 0.009] | [0.012, 0.017] | [-0.022, -0.018] | [-0.010, -0.007] |  |
| AME Model 2 – AME Model 1 | {-0.002}*** | {-0.003}*** | {-0.003}*** | {0.008}*** | {0.000} |  |
| Observations | 247,234 |  |  |  |  |  |
| Individuals | 36,312 |  |  |  |  |  |

*Note.* Main entries are AME’s, 95% confidence intervals using clustered standard errors by individual in brackets. In curly brackets we report tests for the difference in AME’s across the models, following the procedure in Mize, Doan and Long (2019). Cognitive ability is our standardized age-effect-free general cognitive ability factor. Sociodemographic controls include age (in quadratic form); gender; marital status; the number of dependent children in the household; the square root of household size; and region of residence and Wave controls. Socioeconomic controls include housing tenure; economic activity; logarithm of monthly household income (which is adjusted by the OECD-modified equivalence scale, deflated by the Consumer Price Index and entered in quadratic form); logarithm of monthly personal income (which is deflated by the Consumer Price Index and entered in quadratic form) and self-assessed general health. Educational attainment controls represent the highest level of attainment. The relative frequency for each category of our dependent variable is as follows: ‘Extreme pessimism’ = 0.07; ‘Moderate pessimism’ = 0.30; ‘Realism’ = 0.26; ‘Moderate optimism’ = 0.29; ‘Extreme optimism’ = 0.09. AME = Average marginal effect.

* p<0.05, ** p<0.01, *** p<0.001.

**Table S5.** Multinomial logistic regressions measuring the relationship between cognitive ability and the 5-point unrealistic optimism scale. Financial realizations coded around a ‘no change’ in household income of between -7.5% and 7.5%.

|  | Dependent variable: 5-point unrealistic optimism scale | | | | | |
| --- | --- | --- | --- | --- | --- | --- |
|  | Extreme pessimism | Pessimism | Realism | Optimism | Extreme optimism |  |
| Model 1: Sociodemographic and socioeconomic controls | | | | | |  |
| Cognitive ability | 0.008*** | 0.012*** | 0.016*** | -0.027*** | -0.008*** |  |
|  | [0.007, 0.009] | [0.010, 0.014] | [0.013, 0.018] | [-0.029, -0.025] | [-0.010, -0.007] |  |
| Model 2: Sociodemographic, socioeconomic and educational attainment controls | | | | | |  |
| Cognitive ability | 0.006*** | 0.007*** | 0.015*** | -0.020*** | -0.008*** |  |
|  | [0.004, 0.007] | [0.005, 0.009] | [0.013, 0.018] | [-0.022, -0.018] | [-0.009, -0.007] |  |
| AME Model 2 – AME Model 1 | {-0.002}*** | {-0.005}*** | {-0.000} | {0.007}*** | {0.001}* |  |
| Observations | 247,234 |  |  |  |  |  |
| Individuals | 36,312 |  |  |  |  |  |

*Note.* Main entries are AME’s, 95% confidence intervals using clustered standard errors by individual in brackets. In curly brackets we report tests for the difference in AME’s across the models, following the procedure in Mize, Doan and Long (2019). Cognitive ability is our standardized age-effect-free general cognitive ability factor. Sociodemographic controls include age (in quadratic form); gender; marital status; the number of dependent children in the household; the square root of household size; and region of residence and Wave controls. Socioeconomic controls include housing tenure; economic activity; logarithm of monthly household income (which is adjusted by the OECD-modified equivalence scale, deflated by the Consumer Price Index and entered in quadratic form); logarithm of monthly personal income (which is deflated by the Consumer Price Index and entered in quadratic form) and self-assessed general health. Educational attainment controls represent the highest level of attainment. The relative frequency for each category of our dependent variable is as follows: ‘Extreme pessimism’ = 0.05; ‘Moderate pessimism’ = 0.26; ‘Realism’ = 0.35; ‘Moderate optimism’ = 0.26; ‘Extreme optimism’ = 0.07. AME = Average marginal effect.

* p<0.05, ** p<0.01, *** p<0.001.

**Table S6.** Multinomial logistic regressions measuring the relationship between cognitive ability and the 5-point unrealistic optimism scale. Financial realizations coded around a ‘no change’ in household income of between -12.5% and 12.5%.

|  | Dependent variable: 5-point unrealistic optimism scale | | | | | |
| --- | --- | --- | --- | --- | --- | --- |
|  | Extreme pessimism | Pessimism | Realism | Optimism | Extreme optimism |  |
| Model 1: Sociodemographic and socioeconomic controls | | | | | |  |
| Cognitive ability | 0.006*** | 0.014*** | 0.012*** | -0.024*** | -0.008*** |  |
|  | [0.005, 0.007] | [0.012, 0.016] | [0.010, 0.015] | [-0.026, -0.022] | [-0.009, -0.007] |  |
| Model 2: Sociodemographic, socioeconomic and educational attainment controls | | | | | |  |
| Cognitive ability | 0.005*** | 0.008*** | 0.013*** | -0.019*** | -0.007*** |  |
|  | [0.003, 0.006] | [0.006, 0.010] | [0.011, 0.016] | [-0.021, -0.016] | [-0.009, -0.006] |  |
| AME Model 2 – AME Model 1 | {-0.002}*** | {-0.005}*** | {0.001}** | {0.006}*** | {0.000}* |  |
| Observations | 247,234 |  |  |  |  |  |
| Individuals | 36,312 |  |  |  |  |  |

*Note.* Main entries are AME’s, 95% confidence intervals using clustered standard errors by individual in brackets. In curly brackets we report tests for the difference in AME’s across the models, following the procedure in Mize, Doan and Long (2019). Cognitive ability is our standardized age-effect-free general cognitive ability factor. Sociodemographic controls include age (in quadratic form); gender; marital status; the number of dependent children in the household; the square root of household size; and region of residence and Wave controls. Socioeconomic controls include housing tenure; economic activity; logarithm of monthly household income (which is adjusted by the OECD-modified equivalence scale, deflated by the Consumer Price Index and entered in quadratic form); logarithm of monthly personal income (which is deflated by the Consumer Price Index and entered in quadratic form) and self-assessed general health. Educational attainment controls represent the highest level of attainment. The relative frequency for each category of our dependent variable is as follows: ‘Extreme pessimism’ = 0.04; ‘Moderate pessimism’ = 0.24; ‘Realism’ = 0.41; ‘Moderate optimism’ = 0.25; ‘Extreme optimism’ = 0.06. AME = Average marginal effect.

* p<0.05, ** p<0.01, *** p<0.001.

**Table S7.** Multinomial logistic regressions measuring the relationship between cognitive ability and the 5-point unrealistic optimism scale. Financial realizations constructed from changes in personal income.

|  | Dependent variable: 5-point unrealistic optimism scale | | | | | |
| --- | --- | --- | --- | --- | --- | --- |
|  | Extreme pessimism | Pessimism | Realism | Optimism | Extreme optimism |  |
| Model 1: Sociodemographic and socioeconomic controls | | | | | |  |
| Cognitive ability | 0.007*** | 0.008*** | 0.019*** | -0.027*** | -0.007*** |  |
|  | [0.005, 0.008] | [0.006, 0.010] | [0.017, 0.022] | [-0.029, -0.025] | [-0.009, -0.006] |  |
| Model 2: Sociodemographic, socioeconomic and educational attainment controls | | | | | |  |
| Cognitive ability | 0.004*** | 0.005*** | 0.018*** | -0.021*** | -0.007*** |  |
|  | [0.003, 0.006] | [0.003, 0.007] | [0.016, 0.021] | [-0.023, -0.019] | [-0.008, -0.005] |  |
| AME Model 2 – AME Model 1 | {-0.002}*** | {-0.004}*** | {-0.001}** | {0.006}*** | {0.001}** |  |
| Observations | 247,234 |  |  |  |  |  |
| Individuals | 36,312 |  |  |  |  |  |

*Note.* Main entries are AME’s, 95% confidence intervals using clustered standard errors by individual in brackets. In curly brackets we report tests for the difference in AME’s across the models, following the procedure in Mize, Doan and Long (2019). Cognitive ability is our standardized age-effect-free general cognitive ability factor. Sociodemographic controls include age (in quadratic form); gender; marital status; the number of dependent children in the household; the square root of household size; and region of residence and Wave controls. Socioeconomic controls include housing tenure; economic activity; logarithm of monthly household income (which is adjusted by the OECD-modified equivalence scale, deflated by the Consumer Price Index and entered in quadratic form); logarithm of monthly personal income (which is deflated by the Consumer Price Index and entered in quadratic form) and self-assessed general health. Educational attainment controls represent the highest level of attainment. The relative frequency for each category of our dependent variable is as follows: ‘Extreme pessimism’ = 0.06; ‘Moderate pessimism’ = 0.28; ‘Realism’ = 0.33; ‘Moderate optimism’ = 0.26; ‘Extreme optimism’ = 0.07. AME = Average marginal effect.

* p<0.05, ** p<0.01, *** p<0.001.

**Table S8.** Multinomial logistic regressions measuring the relationship between cognitive ability and the 5-point unrealistic optimism scale. Restricting the sample to those who are observed in at least seven Waves.

|  | Dependent variable: 5-point unrealistic optimism scale | | | | | |
| --- | --- | --- | --- | --- | --- | --- |
|  | Extreme pessimism | Pessimism | Realism | Optimism | Extreme optimism |  |
| Model 1: Sociodemographic and socioeconomic controls | | | | | |  |
| Cognitive ability | 0.008*** | 0.011*** | 0.017*** | -0.027*** | -0.008*** |  |
|  | [0.007, 0.009] | [0.008, 0.013] | [0.014, 0.020] | [-0.030, -0.025] | [-0.010, -0.007] |  |
| Model 2: Sociodemographic, socioeconomic and educational attainment controls | | | | | |  |
| Cognitive ability | 0.005*** | 0.005*** | 0.016*** | -0.019*** | -0.007*** |  |
|  | [0.004, 0.007] | [0.003, 0.008] | [0.013, 0.019] | [-0.022, -0.017] | [-0.009, -0.006] |  |
| AME Model 2 – AME Model 1 | {-0.003}*** | {-0.005}*** | {-0.001}** | {0.008}*** | {0.001}*** |  |
| Observations | 191,176 |  |  |  |  |  |
| Individuals | 20,266 |  |  |  |  |  |

*Note.* Main entries are AME’s, 95% confidence intervals using clustered standard errors by individual in brackets. In curly brackets we report tests for the difference in AME’s across the models, following the procedure in Mize, Doan and Long (2019). Cognitive ability is our standardized age-effect-free general cognitive ability factor. Sociodemographic controls include age (in quadratic form); gender; marital status; the number of dependent children in the household; the square root of household size; and region of residence and Wave controls. Socioeconomic controls include housing tenure; economic activity; logarithm of monthly household income (which is adjusted by the OECD-modified equivalence scale, deflated by the Consumer Price Index and entered in quadratic form); logarithm of monthly personal income (which is deflated by the Consumer Price Index and entered in quadratic form) and self-assessed general health. Educational attainment controls represent the highest level of attainment. The relative frequency for each category of our dependent variable is as follows: ‘Extreme pessimism’ = 0.06; ‘Moderate pessimism’ = 0.28; ‘Realism’ = 0.31; ‘Moderate optimism’ = 0.28; ‘Extreme optimism’ = 0.07. AME = Average marginal effect.

* p<0.05, ** p<0.01, *** p<0.001.

**Table S9.** Pairwise correlations of unrealistic optimism, cognitive ability and the Big-Five

| Variables | (1) | | (2) | | (3) | | (4) | | (5) | | (6) | | (7) | | (8) | | (9) | | (10) | |  |
| --- | --- | --- | --- | --- | --- | --- | --- | --- | --- | --- | --- | --- | --- | --- | --- | --- | --- | --- | --- | --- | --- |
| (1) Extreme pessimism |  | |  | |  | |  | |  | |  | |  | |  | |  | |  | |  |
| (2) Moderate Pessimism | | -.155*** | |  | |  | |  | |  | |  | |  | |  | |  | |  | |
| (3) Realism | -.168*** | | -.419*** | |  | |  | |  | |  | |  | |  | |  | |  | |  |
| (4) Moderate optimism | -.153*** | | -.382*** | | -.413*** | |  | |  | |  | |  | |  | |  | |  | |  |
| (5) Extreme optimism | -.072*** | | -.179*** | | -.194*** | | -.177*** | |  | |  | |  | |  | |  | |  | |  |
| (6) Cognitive ability | -.003 | | -.014*** | | .032*** | | -.008*** | | -.015*** | |  | |  | |  | |  | |  | |  |
| (7) Openness | -.007*** | | -.027*** | | .014*** | | -.007*** | | .038*** | | .178*** | |  | |  | |  | |  | |  |
| (8) Neuroticism | .040*** | | .009*** | | -.003 | | -.014*** | | -.023*** | | -.080*** | | -.128*** | |  | |  | |  | |  |
| (9) Extraversion | -.019*** | | -.013*** | | .004** | | .003 | | .027*** | | .013*** | | .257*** | | -.204*** | |  | |  | |  |
| (10) Conscientious | -.024*** | | -.006*** | | .002 | | .007*** | | .016*** | | .006*** | | .216*** | | -.169*** | | .217*** | |  | |  |
| (11) Agreeableness | -.020*** | | -.001 | | -.002 | | .008*** | | .010*** | | -.066*** | | .202*** | | -.059*** | | .177*** | | .335*** | |  |

*Note.* Sample of 36,312 individuals with 247,234 person-Wave observations. The 5-point unrealistic optimism scale is included as five binary indicators for each category. Cognitive ability is our standardized age-effect-free general cognitive ability factor. Big-Five personality factors are measured using the short 15-item Big-Five inventory (BFI-15). Each trait is based on a level of agreement with three statements, assessed on a seven-point scale. Responses are added across each set of the three statements and then divided by the number of items over which the sum is calculated.

* p<0.05, ** p<0.01, *** p<0.001.

**Table S10.** Multinomial logistic regressions measuring the relationship between cognitive ability and the 5-point unrealistic optimism scale. Controlling for the Big-Five.

|  | Dependent variable: 5-point unrealistic optimism scale | | | | | |
| --- | --- | --- | --- | --- | --- | --- |
|  | Extreme pessimism | Pessimism | Realism | Optimism | Extreme optimism |  |
| Model 1: Sociodemographic, socioeconomic controls and Big-Five controls | | | | | |  |
| Cognitive ability | 0.008*** | 0.012*** | 0.016*** | -0.026*** | -0.010*** |  |
|  | [0.007, 0.009] | [0.009, 0.014] | [0.014, 0.019] | [-0.028, -0.024] | [-0.011, -0.008] |  |
| Model 2: Sociodemographic, socioeconomic, educational attainment and Big-Five controls | | | | | |  |
| Cognitive ability | 0.006*** | 0.007*** | 0.015*** | -0.020*** | -0.009*** |  |
|  | [0.005, 0.007] | [0.005, 0.010] | [0.013, 0.017] | [-0.022, -0.017] | [-0.010, -0.008] |  |
| AME Model 2 – AME Model 1 | {-0.002}*** | {-0.004}*** | {-0.001}*** | {0.007}*** | {0.001}* |  |
| Observations | 247,234 |  |  |  |  |  |
| Individuals | 36,312 |  |  |  |  |  |

*Note.* Main entries are AME’s, 95% confidence intervals using clustered standard errors by individual in brackets. In curly brackets we report tests for the difference in AME’s across the models, following the procedure in Mize, Doan and Long (2019). Cognitive ability is our standardized age-effect-free general cognitive ability factor. Sociodemographic controls include age (in quadratic form); gender; marital status; the number of dependent children in the household; the square root of household size; and region of residence and Wave controls. Socioeconomic controls include housing tenure; economic activity; logarithm of monthly household income (which is adjusted by the OECD-modified equivalence scale, deflated by the Consumer Price Index and entered in quadratic form); logarithm of monthly personal income (which is deflated by the Consumer Price Index and entered in quadratic form) and self-assessed general health. Educational attainment controls represent the highest level of attainment. Big-Five controls include personality traits—Openness, Neuroticism, Extraversion, Conscientiousness and Agreeableness—which are measured using the short 15-item Big-Five inventory (BFI-15). Each trait is based on a level of agreement with three statements, assessed on a seven-point scale. Responses are added across each set of the three statements and then divided by the number of items over which the sum is calculated. Full results are available on request. The relative frequency for each category of our dependent variable is as follows: ‘Extreme pessimism’ = 0.06; ‘Moderate pessimism’ = 0.28; ‘Realism’ = 0.31; ‘Moderate optimism’ = 0.27; ‘Extreme optimism’ = 0.08. AME = Average marginal effect.

* p<0.05, ** p<0.01, *** p<0.001.

**Section B**

Alternative methods of estimating differences in optimism bias for levels of cognitive ability are reviewed here and the corresponding estimates presented. All methods find that those high on cognitive ability are less unrealistically optimistic than those low on cognitive ability.

As a first test, using multinomial logistic regression, we separately estimated the influence of cognitive ability on our 3-point measures of financial expectations and financial realizations (i.e., $E_{iw}$ and $R_{iw+1}$). We reasoned, that if those low on cognitive ability had significantly worse realizations but higher expectations, then it can be concluded that those low on cognitive ability are the most unrealistically optimistic. Table S11 and S12 presents the results from the financial expectation and realization regressions, respectively. For ease of exposition and following convention, we report the average marginal effect (AME) of a one standard deviation increase in our age-effect-free general cognitive ability factor on the probability of observing each of the three outcomes of our dependent variables. As depicted in Tables S11 and S12, in all models, those low on cognitive ability are less likely to have a financial expectation of ‘worse off’ yet they are much more likely to experience a financial realization of ‘worse off”. For instance, from Model 1 of Table S11 those low on cognitive ability (-2 standard deviations from the mean) have a predicted probability of a ‘worse off’ financial expectation of 12.8%, whilst those high on cognitive ability (+2 standard deviations from the mean) have a probability of 18.5%. However, from Model 1 of Table S12 those low on cognitive ability have a predicted probability of a ‘worse off’ financial realization of 44.9%, whilst those high on cognitive ability have a probability of 30.6%. In short, whilst both groups underestimate the probability of negative events, the extent of this underestimation is significantly larger for those low on cognitive ability. Moreover, whilst cognitive ability has a negligible effect on the probability of expecting ‘better off’, those high on cognitive ability are much more likely to experience a financial realization of ‘better off”. It can therefore be concluded that those low on cognitive ability are the most unrealistically optimistic.

As a second test, using multinomial logistic regression, we estimated the influence of cognitive ability on financial realizations, $R_{iw+1}$, controlling for financial expectations, $E_{iw}$. We reasoned, that if cognitive ability has no effect on optimism bias, then the distribution of financial realizations would be the same conditional on expectations. However, for instance, if those high on cognitive ability are found to have significantly better (worse) financial realizations, controlling for financial expectations, then it can be concluded that those high on cognitive ability are the least (most) unrealistically optimistic. As depicted in Table S13, and similar to the results presented in Table S12, in all models those high on cognitive ability are associated with higher financial realizations, controlling for financial expectations. It can therefore be concluded that those low on cognitive ability are the most unrealistically optimistic.

**Table S11.** Multinomial logistic regressions measuring the relationship between cognitive ability and the 3-point financial expectations scale

|  | Dependent variable: 3-point financial expectations scale | | |
| --- | --- | --- | --- |
|  | Worse off | No change | Better off |
| Model 1: Sociodemographic and socioeconomic controls | | | |
| Cognitive ability | 0.014*** | -0.017*** | 0.003* |
|  | [0.012, 0.017] | [-0.021, -0.014] | [0.000, 0.006] |
| Model 2: Sociodemographic, socioeconomic and educational attainment controls | | | |
| Cognitive ability | 0.012*** | -0.011*** | -0.001 |
|  | [0.009, 0.015] | [-0.014, -0.008] | [-0.004, 0.002] |
| AME Model 2 – AME Model 1 | {-0.002}*** | {0.006}*** | {-0.004{*** |
| Observations | 247,234 |  |  |
| Individuals | 36,312 |  |  |

*Note.* Main entries are AME’s, 95% confidence intervals using clustered standard errors by individual in brackets. In curly brackets we report tests for the difference in AME’s across the models, following the procedure in Mize, Doan and Long (2019). Cognitive ability is our standardized age-effect-free general cognitive ability factor. Sociodemographic controls include age (in quadratic form); gender; marital status; the number of dependent children in the household; the square root of household size; and region of residence and Wave controls. Socioeconomic controls include housing tenure; economic activity; logarithm of monthly household income (which is adjusted by the OECD-modified equivalence scale, deflated by the Consumer Price Index and entered in quadratic form); logarithm of monthly personal income (which is deflated by the Consumer Price Index and entered in quadratic form) and self-assessed general health. Educational attainment controls represent the highest level of attainment. The relative frequency for each category of our dependent variable is as follows: ‘Worse off’= 0.16; ‘No change’= 0.63; ‘Better off’ = 0.22. AME = Average marginal effect.

* p<0.05, ** p<0.01, *** p<0.001.

**Table S12.** Multinomial logistic regressions measuring the relationship between cognitive ability and the 3-point financial realizations scale

|  | Dependent variable: 3-point financial realizations scale | | |
| --- | --- | --- | --- |
|  | Worse off | No change | Better off |
| Model 1: Sociodemographic and socioeconomic controls | | | |
| Cognitive ability | -0.036*** | 0.010*** | 0.025*** |
|  | [-0.038, -0.034] | [0.008, 0.013] | [0.023, 0.028] |
| Model 2: Sociodemographic, socioeconomic and educational attainment controls | | | |
| Cognitive ability | -0.028*** | 0.013*** | 0.015*** |
|  | [-0.030, -0.026] | [0.011, 0.015] | [0.013, 0.017] |
| AME Model 2 – AME Model 1 | {0.008}*** | {0.003}*** | {-0.010}*** |
| Observations | 247,234 |  |  |
| Individuals | 36,312 |  |  |

*Note.* Main entries are AME’s, 95% confidence intervals using clustered standard errors by individual in brackets. In curly brackets we report tests for the difference in AME’s across the models, following the procedure in Mize, Doan and Long (2019). Cognitive ability is our standardized age-effect-free general cognitive ability factor. Sociodemographic controls include age (in quadratic form); gender; marital status; the number of dependent children in the household; the square root of household size; and region of residence and Wave controls. Socioeconomic controls include housing tenure; economic activity; logarithm of monthly household income (which is adjusted by the OECD-modified equivalence scale, deflated by the Consumer Price Index and entered in quadratic form); logarithm of monthly personal income (which is deflated by the Consumer Price Index and entered in quadratic form) and self-assessed general health. Educational attainment controls represent the highest level of attainment. The relative frequency for each category of our dependent variable is as follows: ‘Worse off’= 0.37; ‘No change’= 0.23; ‘Better off’ = 0.40. AME = Average marginal effect.

* p<0.05, ** p<0.01, *** p<0.001.

**Table S13.** Multinomial logistic regressions measuring the relationship between cognitive ability and the 3-point financial realizations scale conditional on financial expectations

|  | Dependent variable: 3-point financial realizations scale | | |
| --- | --- | --- | --- |
|  | Worse off | No change | Better off |
| Model 1: Sociodemographic and socioeconomic controls | | | |
| Cognitive ability | -0.036*** | 0.011*** | 0.026*** |
|  | [-0.038, -0.034] | [0.008, 0.013] | [0.023, 0.028] |
| Model 2: Sociodemographic, socioeconomic and educational attainment controls | | | |
| Cognitive ability | -0.029*** | 0.013*** | 0.015*** |
|  | [-0.031, -0.026] | [0.011, 0.015] | [0.013, 0.018] |
| AME Model 2 – AME Model 1 | {0.008}*** | {0.002}*** | {-0.010}*** |
| Observations | 247,234 |  |  |
| Individuals | 36,312 |  |  |

*Note.* Main entries are AME’s, 95% confidence intervals using clustered standard errors by individual in brackets. In curly brackets we report tests for the difference in AME’s across the models, following the procedure in Mize, Doan and Long (2019). Cognitive ability is our standardized age-effect-free general cognitive ability factor. All regressions included control variables for financial expectations. Sociodemographic controls include age (in quadratic form); gender; marital status; the number of dependent children in the household; the square root of household size; and region of residence and Wave controls. Socioeconomic controls include housing tenure; economic activity; logarithm of monthly household income (which is adjusted by the OECD-modified equivalence scale, deflated by the Consumer Price Index and entered in quadratic form); logarithm of monthly personal income (which is deflated by the Consumer Price Index and entered in quadratic form) and self-assessed general health. Educational attainment controls represent the highest level of attainment. The relative frequency for each category of our dependent variable is as follows: ‘Worse off’= 0.37; ‘No change’= 0.23; ‘Better off’ = 0.40. AME = Average marginal effect.

* p<0.05, ** p<0.01, *** p<0.001.
